# Supplementary material for: Recent Trends in Dietary Habits of the Italian Population: Potential Impact on Health and the Environment
Source: Nutrients. 2021 Jan 31;13(2):476. doi: 10.3390/nu13020476 (PMC7911362; doi:10.3390/nu13020476)
Supplement: Supplementary file 1 [file nutrients-13-00476-s001.pdf]

Supplemental Table 1. Crude data without waste correction for all food groups and for each year of the observation period

	**2000**	**2001**	**2002**	**2003**	**2004**	**2005**	**2006**	**2007**	**2008**	**2009**	**2010**	**2011**	**2012**	**2013**	**2014**	**2015**	**2016**	**2017**
**Fruit**	401	366	393	367	424	421	442	442	426	478	418	394	355	382	331	355	324	310
**Vegetables**	547	447	404	485	548	509	447	439	427	490	415	404	378	364	358	378	376	367
**Legumes**	15	15	15	15	15	15	15	15	15	15	15	13	14	15	14	14	14	14
**Nuts**	17	18	19	17	20	20	23	21	21	20	22	23	19	21	19	20	23	23
**Potatoes**	118	113	110	102	113	108	108	108	103	110	110	106	105	105	98	100	99	98
**Cereals**	443	447	444	442	437	428	430	429	428	433	424	425	430	433	431	444	444	441
**Beef meat**	68	66	66	68	64	65	67	66	63	65	63	59	57	51	53	47	46	43
**Pork meat**	103	109	109	110	109	106	110	112	107	109	115	110	109	110	101	107	105	120
**Poultry**	52	50	49	41	43	42	37	43	47	48	48	50	52	51	51	51	51	52
**Fish**	62	67	65	67	66	69	72	71	69	69	71	72	70	70	77	81	82	82
**Eggs**	34	33	32	32	32	32	30	32	32	35	32	32	33	37	33	31	32	32
**Milk and dairies**	735	741	737	718	717	742	738	707	734	708	706	714	700	676	686	661	618	626
**Animal Fat**	37	37	37	35	35	36	37	37	38	35	35	34	37	35	16	16	15	15
**Tropical Oils**	4	4	4	5	4	4	4	5	6	8	9	10	10	12	10	10	10	10
**Non-tropical Oils**	64	64	65	66	62	59	66	67	66	62	64	65	63	60	56	56	58	58
**Sugars**	85	87	86	86	88	87	82	81	81	81	82	84	86	88	90	86	89	89
